# Direct pixel to pixel principal strain mapping from tagging MRI using end to end deep convolutional neural network (DeepStrain)

**DOI:** 10.1038/s41598-021-02279-y

**Published:** 2021-11-26

**Authors:** Khaled Z. Abd-Elmoniem, Inas A. Yassine, Nader S. Metwalli, Ahmed Hamimi, Ronald Ouwerkerk, Jatin R. Matta, Mia Wessel, Michael A. Solomon, Jason M. Elinoff, Ahmed M. Ghanem, Ahmed M. Gharib

**Affiliations:** 1 Biomedical and Metabolic Imaging Branch, National Institute of Diabetes and Digestive and Kidney Diseases (NIDDK), National Institutes of Health, 10 Center Drive, Bldg. 10, CRC, Rm. 3-5340, Bethesda, MD 20892 USA; 2 Systems and Biomedical Engineering Department, Faculty of Engineering, Cairo University, Cairo, Egypt; 3 Cardiovascular Branch of the National Heart, Lung, and Blood Institute (NHLBI), NIH, Bethesda, MD USA; 4 Critical Care Medicine Department, NIH Clinical Center, Bethesda, MD USA

**Keywords:** Biomedical engineering, Translational research, Cardiomyopathies, Imaging techniques, Design, synthesis and processing, Mathematics and computing

## Abstract

Regional soft tissue mechanical strain offers crucial insights into tissue's mechanical function and vital indicators for different related disorders. Tagging magnetic resonance imaging (tMRI) has been the standard method for assessing the mechanical characteristics of organs such as the heart, the liver, and the brain. However, constructing accurate artifact-free pixelwise strain maps at the native resolution of the tagged images has for decades been a challenging unsolved task. In this work, we developed an end-to-end deep-learning framework for pixel-to-pixel mapping of the two-dimensional Eulerian principal strains $\varvec{{\varepsilon }}_{\boldsymbol{p1}}$ and $\varvec{{\varepsilon }}_{\boldsymbol{p2}}$ directly from 1-1 spatial modulation of magnetization (SPAMM) tMRI at native image resolution using convolutional neural network (CNN). Four different deep learning conditional generative adversarial network (cGAN) approaches were examined. Validations were performed using Monte Carlo computational model simulations, and in-vivo datasets, and compared to the harmonic phase (HARP) method, a conventional and validated method for tMRI analysis, with six different filter settings. Principal strain maps of Monte Carlo tMRI simulations with various anatomical, functional, and imaging parameters demonstrate artifact-free solid agreements with the corresponding ground-truth maps. Correlations with the ground-truth strain maps were *R* = 0.90 and 0.92 for the best-proposed cGAN approach compared to *R* = 0.12 and 0.73 for the best HARP method for $\varvec{{\varepsilon }}_{\boldsymbol{p1}}$ and $\varvec{{\varepsilon }}_{\boldsymbol{p2}}$, respectively. The proposed cGAN approach's error was substantially lower than the error in the best HARP method at all strain ranges. In-vivo results are presented for both healthy subjects and patients with cardiac conditions (Pulmonary Hypertension). Strain maps, obtained directly from their corresponding tagged MR images, depict for the first time anatomical, functional, and temporal details at pixelwise native high resolution with unprecedented clarity. This work demonstrates the feasibility of using the deep learning cGAN for direct myocardial and liver Eulerian strain mapping from tMRI at native image resolution with minimal artifacts.

## Introduction

Tagging MRI (tMRI) has been used for imaging soft tissue deformation in several human organs, including the heart, liver, brain, and tongue^1–4^. A set of virtual noninvasive markers, called tags, are generated into the target tissue. These tags follow tissue motion and deformation. A cinematic data set (cine) is then acquired during the cardiac cycle to demonstrate the motion in the imposed tags and the underlying tissue deformation during that period. The tags could be lines or grids of waveforms ranging from simple sinusoids to square waveform patterns^5–8^.

Numerous frameworks were proposed for tMRI analysis in both the spatial and the k-space domains. Methods can also be categorized as feature-based, model-based, or bandpass filtering-based. Feature-based methods track sparse tag features and apply interpolation to create a continuous displacement or strain field map. Deformable modeling approaches involve fitting tMRI data to a mathematical model by minimizing certain energy functions. Bandpass filtering methods apply various families of filters to obtain continuous deformation maps from tMRI data. Harmonic phase (HARP) method is a conventional, widely acceptable, and validated k-space bandpass filtering tMRI analysis framework^9^. HARP analyzes the displacement information encoded in the off-center harmonic spectral peaks and obtains the two-dimensional (2D) displacement maps later used to calculate the associated 2D strain maps.

Many methods were proposed to improve HARP computations and tMRI sequences to enhance HARP performance^10,11^. Comprehensive reviews on tMRI analysis methods have been previously published^12^. Limitations of these methods include limited strain field resolution and restricted dynamic range due to the intrinsic broad-band filtering utilized in the process, extensive regularization, and interpolation of the sparse results. These limitations constrain the applicability of the obtained strain maps to large late-stage anomalies that are likely detectable by other clinical surrogates or more direct imaging methods. The performance of all these methods is also highly dependent on and sensitive to a large set of parameters. These parameters include model settings, energy minimization, regularization, interpolation, filtering size, shape, or orientation. This sensitivity to parameter selection limits the generalizability of these techniques and increases the likely error in interpreting the results.

Recently, Ferdian et al.^13^ developed a deep learning-based method to obtain radial and circumferential Green (Lagrangian) strains of the left ventricle (LV) at three short-axis cross-sections; basal, mid, and apical from grid tMRI cine datasets. Strain computations were performed by tracking specific landmarks at each cross-section during the cardiac cycle using two convolutional neural networks (CNN) and a recurrent neural network (RNN). Ferdian's approach, however, has the following limitations.

1. The framework is limited to the estimation of radial and circumferential strain maps, beneficial only for visualizing deformation in the short-axis cross-sections of the LV. Their work is inapplicable to situations including other imaging orientations, studying right ventricle strain, or strain in other organs.
2. The framework estimates radial and circumferential strains only at 168 tagging landmarks then interpolates to create a full deformation map, thus generating substantially limited low-resolution maps.
3. The framework processes result in a fixed number of time frames (20-time frames).

Despite the previously extensive work on tMRI analysis, an accurate direct strain mapping at the native resolution with low artifacts is still unachievable. In this work, we developed a tMRI whole image machine-learning framework for the direct estimation of the principal strain maps at native image resolution.

### Background

#### Continuum deformation

Consider a continuum body $D$ in Euclidean Space ${\mathbb{E}}^{3}$ that is moving and deforming. ${D}_{\circ }$ is a reference configuration of $D$; also called the material configuration. $x=x(X,t)$ describes the motion of $D$ at time $t$ where $X$ is the material coordinates, and $x$ is the spatial coordinates of a material element at time $t$. The relation between a material element $dX$ at reference configuration and its spatial corrodents $dx$ at time $t$ is given by

$$dx = x(X+ dX,t) - x(X,t) = \nabla x \, dX$$

where $$\nabla x=\left(\begin{array}{ccc}\frac{{\partial x}_{1}}{{\partial X}_{1}}& \frac{{\partial x}_{1}}{{\partial X}_{2}}& \frac{{\partial x}_{1}}{{\partial X}_{3}}\\ \frac{{\partial x}_{2}}{{\partial X}_{1}}& \frac{{\partial x}_{2}}{{\partial X}_{2}}& \frac{{\partial x}_{2}}{{\partial X}_{3}}\\ \frac{{\partial x}_{3}}{{\partial X}_{1}}& \frac{{\partial x}_{3}}{{\partial X}_{2}}& \frac{{\partial x}_{3}}{{\partial X}_{3}}\end{array}\right)$$.

Let

1 $$dx = F \, dX,$$

where $F= \nabla x$ is the deformation gradient tensor. For incompressible soft tissues, $dx$ can be zero only if $dX$ is zero and the reflection in deformation impossible. Accordingly, ${F}^{-1}$ exists, $\mathrm{det}F>0$ and $F$ could be decomposed into the product of a symmetric tensor and a proper orthogonal tensor as.

$$F= VR,$$

$$dx = VR \, dX,$$

where $V$ is positive definite symmetric tensors, and $R$ is a proper orthogonal tensor.

According to the polar decomposition theorem^14^, $R,$ and $V$ are unique. $R$ represents a pure rotation, and V represents pure stretching or contraction such that.

$$F{F}^{T}=\left(VR\right){(VR)}^{T}=VR{R}^{T}{V}^{T}=V V={V}^{2},$$

2 $$B={V}^{2}=F{F}^{T},$$

where $B$ is left Cauchy-Green tensor.

The Eulerian strain $e$ is defined by

3 $$e ={1/2 }(I-{B}^{-1})$$

The Eulerian strain is calculated directly in the spatial xy-coordinates^14^ and was thus utilized in this work.

Consider a 2D slice, the diagonal elements of the Eulerian strain tensor $e$; ${\varepsilon }_{xx}$ and ${\varepsilon }_{yy}$, represent normal strain in the spatial configuration coordinates, whereas the off-diagonal element ${\varepsilon }_{xy}$ quantifies the shear strain. Since the $e$ tensor is symmetric, this results in two mutually perpendicular eigenvectors $(\boldsymbol{n}_{1},\boldsymbol{n}_{2})$ where $e$ is defined as

4 $$e= \left(\begin{array}{cc}{\varepsilon }_{xx}& {\varepsilon }_{xy}\\ {\varepsilon }_{xy}& {\varepsilon }_{yy}\end{array}\right)={\left(\begin{array}{cc}{\varepsilon }_{p1}& 0\\ 0& {\varepsilon }_{p2}\end{array}\right)}_{\left\{{{\varvec{n}}}_{{\varvec{i}}}\right\}}$$

Eigenvectors $(\boldsymbol{n}_{1},\boldsymbol{n}_{2})$ are the principal directions representing the directions of the maximum stretching and maximum contraction, respectively, and the associated eigenvalues, ${\varepsilon }_{p1}$ and ${\varepsilon }_{p2}$, are the magnitudes of the corresponding principal strains.

#### Generative adversarial network (GAN)

The Generative adversarial network (GAN) first proposed by Goodfellow et al.^15^ generates realistic synthetic samples indistinguishable from the training dataset. Its architecture consists of two competitive neural networks; a generator (G) and a discriminator (D). GANs utilize a competing approach to train the two networks simultaneously. The generator maps random noise Z into synthetic data G(Z). The discriminator as a binary classifier differentiates between the real and generated data and is trained to distinguish between authentic and generated data accurately. On the other hand, the generator is trained to generate more realistic data, reducing the discriminator's ability to recognize it as fake data. The training objective functions of D and G are defined by^15^;

5 $$\underset{D}{\mathcal{L}}= \underset{D}{\mathrm{max}}{\mathbb{E}}_{{X}_{r}\sim {P}_{r}}[\mathrm{log}D({X}_{r})]+ {\mathbb{E}}_{z\sim {P}_{z}}[1-\mathrm{log}D(G(Z))],$$

6 $$\underset{G}{\mathcal{L}}= \underset{G}{\mathrm{min}} {\mathbb{E}}_{z\sim {P}_{z}}[1-\mathrm{log}D(G(Z))] ,$$

${X}_{r}, {P}_{r},$ and ${P}_{z}$ represent the actual data sample, actual data distribution, and the input noise distribution, respectively. The state-of-the-art performance achieved by GANs in many data generation applications encouraged its adoption in medical imaging applications such as image reconstruction, registration, segmentation, image synthesis, among others^16^.

### Strategy outline

The proposed machine-learning framework utilizes conditional generative adversarial network (cGAN) for estimating the full pixelwise Eulerian principal strain maps, as in Eq. (4), associated with the corresponding tMRI images with 1-1 SPAMM sinusoidal line tags. The estimated strain maps are obtained at native image resolution with high accuracy and very low artifacts. The proposed network was trained using three different training approaches to investigate the relative influence of each subnetwork on the stability and performance of the total cGAN, as shown in Table 1. The first approach, the D-Only approach, calculates the generator's loss function^15^ using the discriminator's output only. The second approach, the D–G approach, uses both the generator's and the discriminator's outputs in the generator's loss function. We employed two versions of D–G; D–G1 and D–G2, with the D–G1 version has less contribution of the discriminator's output in the generator's loss function than D–G2. In the third cGAN, the G-Only approach, the generator's loss function involved only the generator's output. In that scheme, the discriminator error was not included in the generator's loss function. The mathematical derivations, networks architecture, and training approaches of the proposed framework are described in further details later in the “Methods” section after “Results” and “Discussion” sections.

**Table 1:** ${\alpha }_{1}$ and ${\alpha }_{2}$ values for different learning approaches.

	Discriminator weight ${\alpha }_{1}$	Generator weight ${\alpha }_{2}$
D-Only	0	1
G-Only	1	0
D-G1	${10}^{4}$	1
D-G2	${10}^{3}$	1

## Results

### Experiments

The four GAN schemes with different loss minimization criteria were assessed. For comparison, the HARP technique was considered as a representative of filter-based tMRI analysis methods. A bandpass filter centered at the positive spectral harmonic peak was utilized to isolate the harmonic peak with six different full widths at half maximum (FWHM) being studied, ranging from 8 to 28 pixels wide.

The experiments were performed using simulated and in-vivo datasets. In the simulated datasets, the performances of all methods were compared pixelwise, once against the ground-truth strain maps and once more against the maps from the best-performing GAN method. In the in-vivo cases, the performances of all methods were compared against the best GAN method. Correlation and scatter plots, and error histogram bar charts were calculated for assessment and demonstration.

Additionally, an expert in tMRI analysis and interpretation visually evaluated the extent of artifacts in 100 randomly chosen strain map square patches. Each one is of size 5 mm × 5 mm during early systole low-strain frames and again in the late systole frames when greater strains typically occur. Scores from 1 to 4 were assigned from minimal to severe map distortion such that minimal represents patches with indistinguishable or insignificant artifacts that are hardly seen with a peak magnitude likely within ± 0.02 over the actual strain value. Patches with mild artifacts are shown with mild ripple interference patterns with the ability to determine the correct strain with a peak magnitude likely within ± 0.05 over the actual strain value. Moderate artifacts are presented with substantial visual ripple patterns that will likely obstruct the ability to distinguish the actual strain value and with a ripple peak magnitude of around ± 0.1 above the actual strain values. Severe artifacts are represented by substantial ripple patterns greater than ± 0.1 in magnitude and completely deter the strain map worthless.

### Simulated datasets

The synthetic datasets were generated using an in-house developed tMRI simulator. A total of 1362 images from 200 simulated tMRI cines were included in the experiments. The synthetic datasets simulate the deformation in the heart and liver at the heart's short-axis view. The simulated tagging lines were imposed on in-vivo MRI images previously acquired using three-dimensional Turbo Field Echo (3D-TFE) or cine balanced Fast Field Echo (bFFE) sequences. Broad range of signal-to-noise ratios, geometrical parameters, and deformation variations were used while generating the datasets. A full description of the in-house simulator and the synthetic datasets' parameters will be provided in the Methods section.

### In-vivo dataset

#### Data acquisition

Data were collected from healthy adult subjects (N = 3) and patients with pulmonary artery hypertension PAH (N = 3) using a 3.0-Tesla Siemens Magnetom Verio scanner (Siemens Medical Solutions, Erlangen, Germany). All in-vivo scans were approved by the National Institutes of Health (NIH) institutional review board (IRB) with necessary informed consent obtained from all subjects before the scans. All experiments were performed in accordance with relevant named guidelines and regulations. Each subject underwent tMRI scans of the heart's short-axis view using a 1-1 SPAMM segmented gradient-recalled-echo (GRE) sequence. Horizontal and vertical tags were applied and acquired separately. Typical imaging parameters used were: repetition time/echo time (TR/TE) = 42 ms/2.5 ms, field of view (FOV) = 300 mm, matrix size of 192 × 108 for a voxel size resolution of 2.1 × 1.6 mm, slice thickness = 7 mm, radio-frequency (RF) excitation flip angle = 13°, 8 segments/frame, and 17 frames/cine.

In-vivo datasets were all used for testing the performance of different methods. The 204 in-vivo strain maps were compared pixelwise to best-performing deep learning method that has the minimum validation error.

#### Preprocessing

Four parameters in actual tMRI were not included in the simulations and could become sources of substantial error if not considered in the preprocessing, namely, tag spacing, pixel resolution, rectified-sinusoidal tagging, and field inhomogeneity. In tMRI, the appreciated strain amount is relative to both tag spacing and pixel resolution. Consequently, we chose the tag spacing for network training to be 8 mm, and pixel spacing to be 1.1 mm/pixel. In actual imaging, the tag spacing may be set to 8 mm, but pixel resolution is often subject to multiple conflicting settings. Therefore, the first preparation step was to resample the input images to a resolution of 1.1 mm/pixel. Additionally, since the network configuration was only trained on signed sinusoidal tagging, the rectified sinusoidal tagging in some frames of typical datasets has false additional tag lines that lead to incorrect strain maps. To avoid that, the rectified sinusoidal tagging was reverted to its original non-rectified signed-magnitude pattern using the phase information of the complex-valued datasets.

### Experimental results

All four investigated GAN schemes with different loss functions converged to a stable model except the D-Only loss scheme, which was later excluded from the performance analysis and further comparison. The G-Only scheme demonstrated the best performance qualitatively and quantitatively, as shown in the following sections.

#### Qualitative demonstration

**Figure 1:**
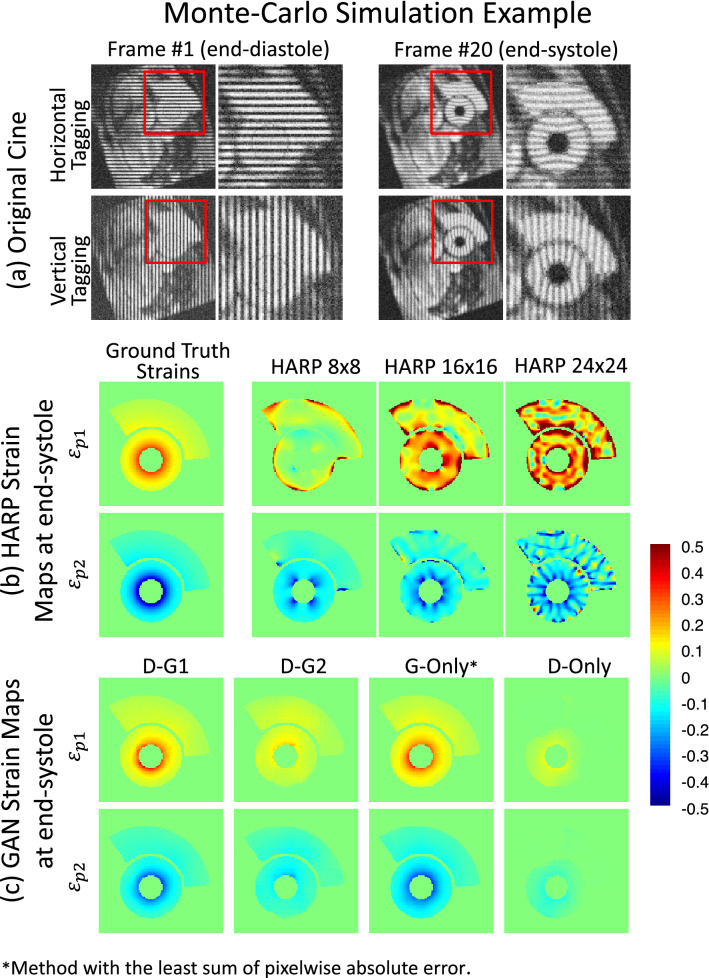
An example of the Monte-Carlo simulated datasets and the corresponding principal strain maps. The principal Eulerian strain maps were estimated using three HARP and four GAN-based methods for the end-systole frame of a simulated dataset. The line tagging images of the end-diastole and end-systole time frames are shown in (**a**). In (**b**), the columns from left to right show the ground truth and the strain maps from the various HARP techniques. Finally, in (**c**), the resulting strain maps from our proposed cGAN techniques are displayed.

Figure 1 demonstrates the computational phantom end-systolic principal Eulerian strains ${\varepsilon }_{p1}$ and ${\varepsilon }_{p2}$ mapped using the GAN and HARP methods with various parameter settings. TMRI data at end-diastole and end-systole are shown in Fig. 1a. Note the end-systole bending of the taglines representing LV contraction and liver deformation. Figure 1b shows the ground-truth strain maps and the HARP strain maps at HARP filter FWHM of 8, 16, and 24 pixels. At FWHM of 8, HARP produces smoothed underestimated strain maps with artifacts near the edges. With wider filters, spatial strain details and variations begin to show up in ${\varepsilon }_{p2}$ and ${\varepsilon }_{p1}$, however, at the expense of more profound and widespread artifacts. On the contrary, ${\varepsilon }_{p1}$ and ${\varepsilon }_{p2}$ maps using GAN methods G-Only and D-G1 in Fig. 1c are substantially close in resemblance to the ground-truth maps without any noticeable difference, with correlation coefficients (R) for ${\varepsilon }_{p1}$ and ${\varepsilon }_{p2}$ of 0.92 and 0.90 in the G-Only case, and 0.898 and 0.913 in the case of D–G1, respectively. The GAN network with the D–G2 error function significantly underestimated strain maps and correlation coefficients R = 0.572 and 0.589, for ${\varepsilon }_{p1}$ and ${\varepsilon }_{p2}$, respectively. Meanwhile, HARP with filter size $16\times 16$ provided the best HARP ${\varepsilon }_{p2}$ estimation (R = 0.734) while R was insignificant (R = 0.055) for ${\varepsilon }_{p1}$.

**Figure 2:**
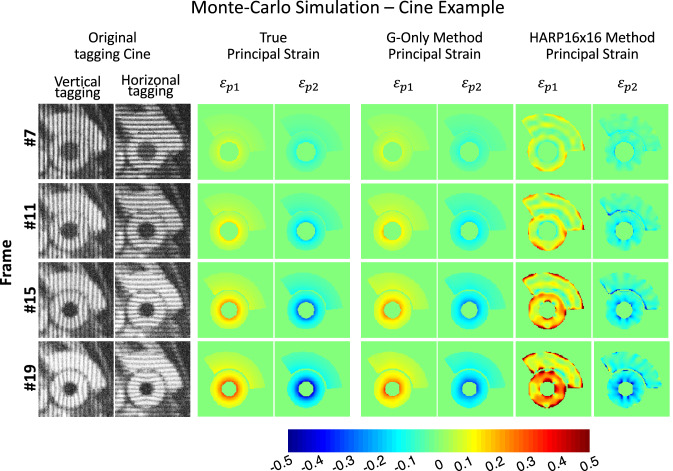
Cine Monte-Carlo simulation strain mapping using GAN versus HARP. G-Only is the best performing scheme among the proposed GAN methods, and HARP $16\times 16$, the best HARP method.

Figure 2 demonstrates a simulated case comparison between the best-performing GAN scheme (G-Only) versus the best-performing HARP (filter size $16\times 16$). The figure further emphasizes the consistent performance of the GAN G-Only method for both ${\varepsilon }_{p1}$ and ${\varepsilon }_{p2}$ versus HARP, in which an increasing amount of artifact in ${\varepsilon }_{p1}$ are observed as ${\varepsilon }_{p1}$ increases whereas ${\varepsilon }_{p2}$ suffers mainly from underestimation. ${\varepsilon }_{p2}$ appears to suffer from some artifacts but to a lesser extent.

**Figure 3:**
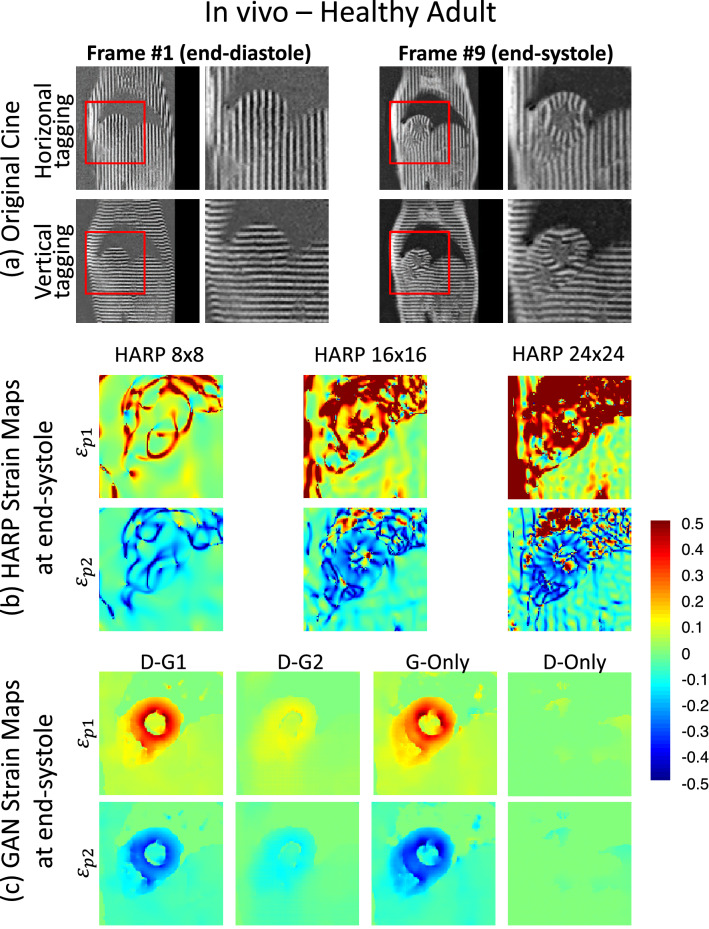
In-vivo principal strain maps at end-systole and end-diastole. Principal strain maps were calculated of the MR tagging dataset of a healthy adult using HARP and directly mapped using the proposed GAN architecture methods. Raw line tagging images of the end-diastole and end-systole time frames are shown in (**a**). HARP technique strain maps are displayed in (**b**) using various parameters. Finally, the resulting strain maps from our proposed GAN techniques are demonstrated in (**c**).

**Figure 4:**
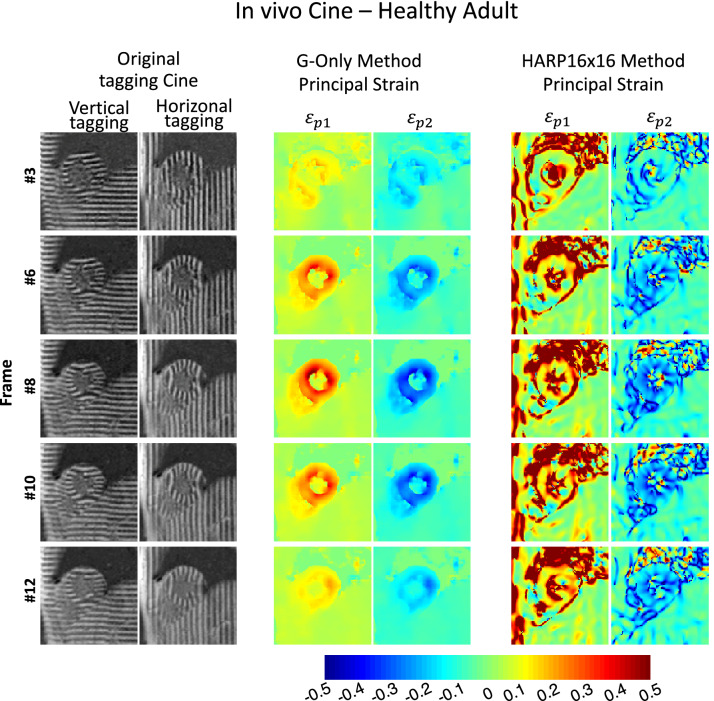
In-vivo cine demonstration of the strain maps generated by G-Only versus HARP in a healthy subject. Compared to HARP and throughout the cardiac cycle, G-Only GAN architecture generated high-resolution artifact-free strain maps with time-progression of strain in both right and left ventricles, and the liver are perceived. An additional movie file shows this in more detail [see Additional file 2].

**Figure 5:**
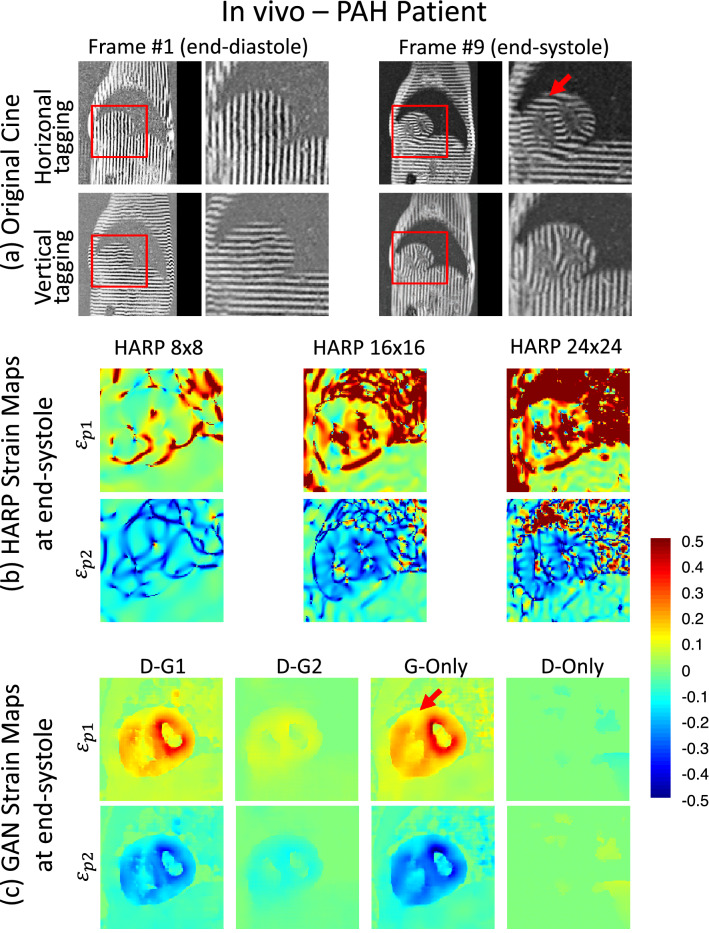
In-vivo demonstration of the strain maps generated by G-Only versus HARP in a PAH patient. (**a**) Raw tagging images of the end-diastole and end-systole time frames. (**b**) Various HARP-based strain maps. (**c**) GAN-based strain maps with different cost functions. High-resolution boundaries of the enlarged left and right ventricles are visible, and strain characteristics of PAH disease such as higher strain magnitude and low strain at the RV-LV insertion point (red arrow).

**Figure 6:**
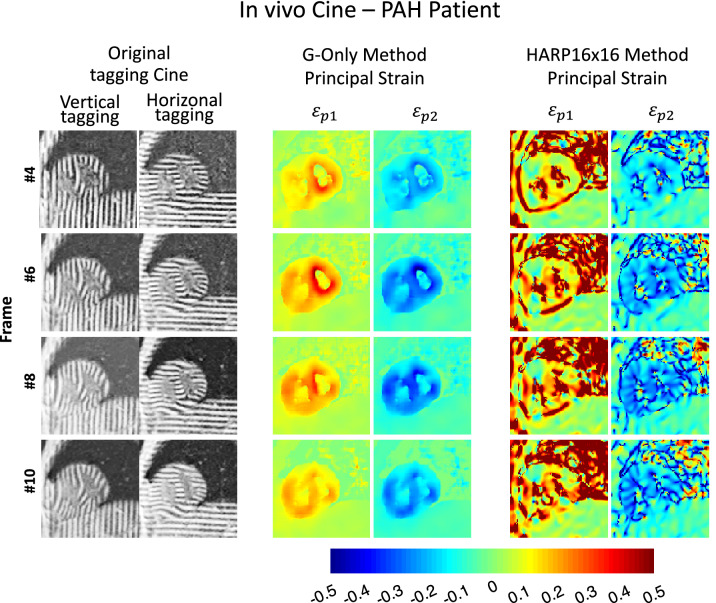
In-vivo cine demonstration of the strain maps generated by G-Only versus HARP in a PAH patient. Throughout the cardiac cycle, and compared to HARP, the G-Only GAN network produced the principal strain maps at the original image native resolution with crisp anatomical boundary details and without artifacts. An additional movie file shows this in more detail [see Additional file 3].

Figures 3 and 4 show the snapshot and cine results from a healthy subject, respectively, whereas Figs. 5 and 6 demonstrate the corresponding results from a PAH patient. Video versions of these demonstrations are available in the supplementary files 1 (Healthy_Adult_Demo) and (2) PAH_Patient_Demo. Among the HARP methods, FWHM of 8 pixels gave the smoothest maps, yet artifacts are still present at the boundaries. These artifacts were aggravated further with larger filter sizes. Meanwhile, G-Only and D–G1 gave substantially higher fidelity strain maps at an unprecedented native acquisition resolution of 1.1 mm.

Notice the ability to generate artifact-free strain maps, both ${\varepsilon }_{p1}$ and ${\varepsilon }_{p2}$, while maintaining the sharp organ boundaries in both the left and right ventricles. This GAN-based superior strain mapping at a native resolution is persistent throughout the cardiac cycle at all contraction levels, as demonstrated in Fig. 4. This accomplishment permits the visualization of the fine details of spatial and temporal strain variations in both the left and right ventricles and even in the papillary muscles. On the contrary, HARP $16\times 16$ strain maps suffer from an increasing number of artifacts as the extent of contraction increases.

Considering the strain maps of the PAH patient as depicted in Figs. 5 and 6 and the supplementary movie file, the right ventricular cavity enlargement and wall thickening are distinctly observable, as well as the substantially elevated levels of strain compared to healthy subjects. Notice also the reduced amount of strain in both the interior and posterior septal segments of LV, all typical PAH pathological characteristics. Additionally, using our GAN-based networks with the aid of the resulting native resolution strain maps, it is now possible to visualize any desynchrony between the mechanical wave propagation between LV and RV in greater detail.

Figure 4 and supplementary video 2 (PAH_Patient_Demo) show that RV and LV contraction timings are in remarkable synchrony. Both ventricles reached peak contraction with a minimal delay at frame #8. In the PAH patient, LV and RV reached peak contraction with a considerable delay at frames #6 and #8, respectively, as shown in Fig. 6. This delay demonstrates the ability to visualize the mechanical resistance the RV encounters when pumping the blood through the pulmonary arterial system. These time and spatial detailed physiological characteristics are not recognizable in the corresponding HARP strain maps.

#### Quantitative demonstration

**Figure 7:**
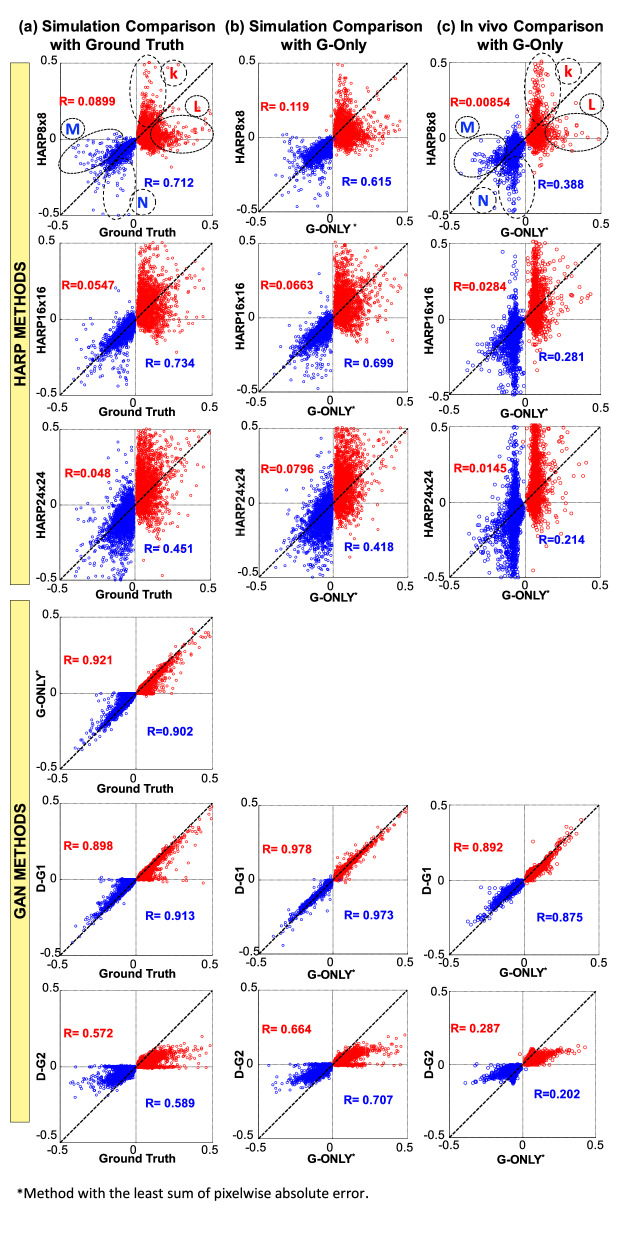
Scatter plot comparisons between various HARP and the proposed GAN-based methods. The first and second principal strains (${\varepsilon }_{p1}$ and ${\varepsilon }_{p2}$) are represented by the red and blue points, respectively. The farther the points deviate coherently from the 45° diagonal identity line, the more the technique over- or under-estimates strain values. K and N regions (dotted elliptic shapes) represent HARP overestimation of ${\varepsilon }_{p1}$ and ${\varepsilon }_{p2}$, respectively, relative to the reference ground-truth strains, whereas L and M represent the corresponding overestimation zones. The comparisons between the different techniques and the ground-truth values for the simulated dataset are shown in (**a**). The comparisons with the G-Only technique for the simulated datasets are shown in (**b**). Finally, the comparisons with the G-Only technique for in-vivo datasets are displayed in panel (**c**).

**Figure 8:**
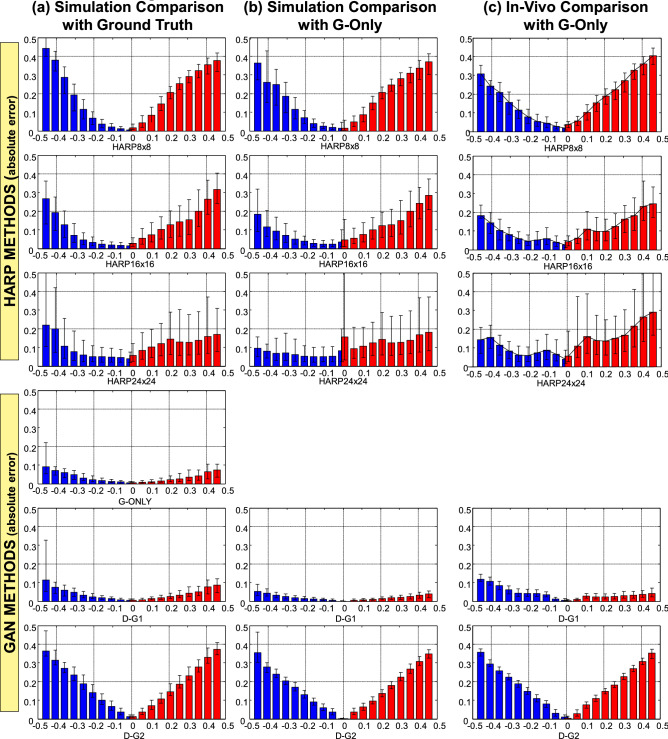
Histograms of the absolute errors in strain measurements for various HARP and proposed GAN methods compared to reference strain values. The absolute errors in the first and second principal strains (${\varepsilon }_{p1}$ and ${\varepsilon }_{p2}$) are represented by the red and blue bars, respectively. Datasets used for analysis and comparison were (**a**) Monte-Carlo simulations' ground-truth maps, (**b**) Monte-Carlo simulations' GAN G-Only maps, and (**c**) In-vivo subjects' GAN G-Only maps.

Quantitative comparisons of all methods are summarized in Figs. 7 and 8. Data of ${\varepsilon }_{p1}$ and ${\varepsilon }_{p2}$ are represented in red and blue colors, respectively. Figure 7a, in the leftmost column, shows the scatter plots and correlations of the HARP and GAN methods versus the ground-truth ${\varepsilon }_{p1}$ and ${\varepsilon }_{p2}$ maps in the computational model test datasets. The dotted 45° lines represent the unity correlation between the ground-truth strain maps and a hypothetically perfect strain mapping method. Points with under-estimated ${\varepsilon }_{p1}$ or ${\varepsilon }_{p2}$ are below or above the unity line and are marked by elliptical regions L or M, respectively. Similarly, points with over-estimated ${\varepsilon }_{p1}$ or ${\varepsilon }_{p2}$ values are marked by the regions K or N, respectively, as shown in Fig. 7.

In the HARP schemes, strain under-estimation, marked in Fig. 7 by the points in and around regions M and L, occurs the most at the smallest filter size because it inherently smooths out noise and artifacts with the cost of under-represents the dynamic range of the underlying strain range. As the filter size increases, the issue of strain underestimation is somewhat mitigated. Nevertheless, strain over-estimation, marked by the points in and around regions K and N, becomes predominant due to harmonic peak interference, filter ringing at the edges, and increased noise-driven artifacts. Strain overestimation is the least when using the smallest filter size and worsens as filter size increases.

Meanwhile, for the GAN schemes, the highest ${\varepsilon }_{p1}$ correlation, R = 0.921, was found with the G-Only approach, whereas the highest ${\varepsilon }_{p2}$ correlation, R = 0.913, was given by the D–G1 approach. The G-Only approach gave the best overall performance. The comparisons between HARP, D–G1, and D–G2 performances to the best-performing GAN, the G-Only method, in both simulation datasets and in-vivo data are shown in Fig. 7b,c, respectively. Note that the same under-estimation and over-estimation patterns in the HARP schemes are also apparent. D–G1 was still highly correlated with the G-Only method in both the simulation and in-vivo settings.

Finally, Fig. 8 shows the histograms of the absolute errors in estimated strain compared to the ground-truth values, Fig. 8a, compared to the G-Only method in simulated datasets, Fig. 8b, and compared to the G-Only method in-vivo, Fig. 8c. The strain range was [0, 0.5] and [− 0.5, 0] for ${\varepsilon }_{p1}$ and ${\varepsilon }_{p2}$, respectively. Each bar represents the median and interquartile range (IQR) of the absolute error in a strain subrange of 0.05. The G-Only method exhibited the lowest absolute error and variation in all subranges among all considered schemes, whereas the highest errors were seen when using HARP with filter size $8\times 8$. As measured by the IQR, enormous fluctuations in error were seen in HARP using filter size $24\times 24$. Results of the comparisons against the G-Only method are similar to those of the comparisons against the ground-truth maps and were consistent in both simulation and in-vivo.

#### Artifact level scoring and evaluation

Table 2 demonstrates the level of artifacts found in each experimented method in both early systole and late systole in one hundred randomly chosen 5 mm × 5 mm square region of interest patches as determined by a coauthor with over 15 years of experience in analyzing tMRI data and blinded to the method used in the analysis. D–G1 and G-Only methods had over 93% of the patches with minimal or unrecognized artifacts, and only around 7% of the patches had mild strain artifacts in the range of ± 0.05.

**Table 2:** Subjective evaluation of the level of artifacts in DL versus HARP methods in 100 randomly chosen 5 mm × 5 mm image patches during (a) early systole and (b) late systole frames.

	Artifact level				
	HARP			Deep learning	
	HARP8 × 8	HARP16 × 16	HARP24 × 24	D-G1	G-Only
**(a) Early systole**					
ε_p1_					
Minimal < 0.02	56	22	4	93	91
Mild < 0.05	21	27	22	7	9
Moderate < 0.1	16	23	28	0	0
Severe > 0.1	7	28	46	0	0
ε_p2_					
Minimal < 0.02	56	27	6	94	93
Mild < 0.05	20	33	22	6	7
Moderate < 0.1	15	23	33	0	0
Severe > 0.1	8	17	39	0	0
**(b) Late systole**					
ε_p1_					
Minimal	21	6	1	93	93
Mild	33	12	4	7	7
Moderate	28	33	18	0	0
Severe	18	49	77	0	0
ε_p2_					
Minimal	27	16	4	93	94
Mild	25	28	24	7	6
Moderate	32	35	37	0	0
Severe	16	21	35	0	0

Meanwhile, HARP methods suffered from substantially more and higher amounts of artifacts. For example, in early systole ${\varepsilon }_{p1}$ maps using HARP16 × 16, only 22% of the patches had minimal artifacts, and 27%, 23%, and 28% of the patches suffered from mild, moderate, and severe amounts of artifacts, respectively. HARP late systole and ${\varepsilon }_{p1}$ maps suffered from more artifacts than early systole and ${\varepsilon }_{p2}$ maps, respectively.

## Discussion

Strain calculation from tMRI images has been a challenging problem since its initial development, mainly due to low signal and contrast ratios of the images (SNR, CNR) and their variation throughout the cine, and frequent motion, breathing, and field artifacts. Previously proposed algorithms for tMRI analysis suffered from significant shortcomings that limit their generalization or acceptance into the clinical settings. These shortcomings include sensitivity to parameter selection, image quality, orientation, artifacts, human interaction, and the low resolution of the obtained maps.

To the best of our knowledge, there is no reliable, fully automated technique that provides Eulerian strain maps directly from the original tagged images at the native image resolution. To overcome the challenges mentioned above, this work utilized GAN and deep learning to fully automate the direct mapping of the Eulerian principal strains at the native image resolution from line-tagged MRI images. Although we focused on the myocardial short-axis images, the algorithms developed in this work are readily applicable to any soft tissue tMRI images such as the brain, the tongue, or the liver, regardless of the organ or section orientation. Different organs and section orientations may have different shapes, strain patterns, and strain value ranges. Therefore, the training data were generated with a large variety in shape geometry, orientations, background, additive and multiplicative noise, fading, and principal strains to improve the proposed techniques' reliability and reduce the effects the anatomical structures may have on the accuracy of the estimated strains.

Moreover, estimating principal strains in place of radial and circumferential strains makes the proposed technique more applicable and beneficial beyond our application to myocardial short-axis cross-sections. The principal directions at any position represent the directions of maximum contraction and stretching. These directions correspond to the macroscopic effective fibers' orientations in muscular tissue as in the myocardium and the force directions in other tissues like the liver or the brain.

High-resolution strain mapping is crucial for visualizing and understanding the subtle spatial strain variations associated with diffused and early infarction, viability, and tumor diseases. It is also important when studying the propagation of the electrophysiological mechanical waves inside the tissue and the desynchrony among different segments. Importantly, high-resolution strain mapping of the right ventricle (RV) may be particularly beneficial for identifying a more severe phenotype of pulmonary vascular disease and enrich clinical trials testing the efficacy of pulmonary vasodilator therapies in patients with the most common causes of pulmonary hypertension (e.g., left heart disease, chronic obstructive pulmonary disease, and interstitial lung disease)^17^.

Pixelwise strain mapping at the native image resolution is hard to achieve using rectangular or other high-order tagging because of the lack of pixelwise motion information. Using sinusoidal patterns helps recognize the small changes at the pixel level, encoded everywhere in the phase component of the sinusoidal pattern. Our algorithm will accept input from any sinusoidal line tagging sequence.

The objective of the study is to obtain the pixelwise mechanical strain map associated with the input tMRI dataset at the same input resolution. Based on the continuum mechanics principles, each tMRI dataset is associated with a single set of ground-truth noise-free, artifact-free principal Eulerian strain maps. The GAN attempts to obtain a set of maps that are as close as possible to ground-truth maps. This is different from many pixel-to-pixel GAN mapping or super-resolution applications, where many solutions are considered correct predictions for any specific input image. In those situations, deep learning-based method usually cannot generate the very correct one every time, caused by its data-driven characteristic. The objective in those applications is to generate output images that have the same probability distribution as the target images. Besides, in our case, the probability of observing high strain in the target strain maps is very low compared to observing low or no strains. These unique characteristics of the problem at hand are reflected in the different effects of the discriminator and generator errors on generator training and final strain estimation accuracy. To investigate these effects, we utilized the four training approaches, D-Only, D-G1, D-G2, and G-Only. As shown in Figs. 1, 3, 5, and 7, D-G2 significantly underestimated the strains, whereas the D-Only scheme barely exhibited any non-zero strains in the strain maps. In contrast, D-G1 and G-Only schemes provided the highest strain estimation accuracy. D-G1 and G-Only have the best correlation coefficients for ${\varepsilon }_{p2}$ (R = 0.913) and ${\varepsilon }_{p1}$ (R = 0.92), respectively. These results demonstrate that reducing the contribution of the generator error in the generator training was associated with underestimating the strains. However, eliminating the discriminator contribution in generator training led to reduced ${\varepsilon }_{p2}$ the correlation coefficient, (R = 0.902) in G-Only, compared to (R = 0.913) in D-G1. More extensive analysis of loss variation during training may shed more light to obtain a network with a higher correlation.

Strain mapping results of the simulated data show that the G-Only and D-G1 provided the minimum absolute errors for all strain ranges and the best correlations with the ground truth. The generated strain maps show no artifacts or inhomogeneity. The contrast between the background and the mathematical phantom regions was clear. In-vivo results show no significant artifacts neither inside the myocardium nor at the boundaries. The resolution was also sufficient to map strains in the RV and LV, even in small structures such as the papillary muscles. In healthy adult datasets, the G-Only scheme's generated strain maps showed healthy myocardial deformation features, including uniform low RV strains and the synchronization between RV and LV contractions. In the PAH patients, the G-Only strain map demonstrated the typical deformation characteristics of the PAH patients, including dilated RV with a high RV muscle strain, low strains at the interior and posterior septum, and delayed RV contractions. All these spatial and temporal strain characteristics can only be recognized in full at high resolution.

Cardiac muscle regional mechanical function is a crucially important factor for clinicians to assess the function and viability of the heart, particularly with MRI that provides this information directly and without any radiation. For decades, using tMRI strain maps has been limited to large regions and compromised in quality and resolution due to the complexity and inefficiency of the classical image tools used for processing and calculations. Obtaining high-resolution strain maps that are artifact-free is challenging using previous tMRI analysis techniques. Feature-based methods typically track the displacement of specific features in the image and use interpolation to create a full yet implicitly low-resolution strain map. While highly sensitive to model parameters, model-based techniques also mandate substantial regularization to allow the underlying energy function to converge. Bandpass filtering methods, including HARP, apply different filters with different filter sizes to calculate regional deformation. Considering HARP as an example, the accuracy of its strain maps may suffer due to noise and artifactual effects if the filter size was too small and from overestimation and substantial amounts and levels of interference artifacts if the filter size was too large. As was shown in Table 2, the severity of artifacts also increases in late systole compared to early systole due to the failure of the HARP fixed-size filter to capture all the contraction information without introducing more artifacts. These typical filtering phenomena were demonstrated in the results.

Meanwhile, utilizing deep CNNs permits an adaptive computationally intensive nonlinear decision-making process far beyond the capabilities of conventional tMRI analysis methods, which only employ one or a few decision-making rules at maximum. These deep CNN characteristics and reformulating the strain estimation to be nonlinear mapping were the key to the success of this work in obtaining high-resolution artifact-free strain maps with unprecedented details that can be appreciated both spatially and temporally. This approach provides a dense strain map that is superior in value to Ferdian's lately published method that computes Lagrangian strain on a limited set of points and only on short-axis views of the heart.

This study, however, has some limitations. The number of in-vivo datasets was limited to six subjects. Data was used only to demonstrate the performance of the proposed technique. While spatial and temporal differences in strain maps between a healthy subject and a PAH patient were demonstrated, more extensive clinical studies are needed to confirm the clinical relevance and applicability of the proposed method. In-vivo data were not used in the training phase. However, the computational phantom provided a vast pool of cases and situations very close to actual data with the advantage of having the ground-truth strain maps available for training rather than the subjective manually obtained maps. Additionally, the performance was examined using in-vivo data from a single MRI machine. Full performance assessment is needed across multiple machines from different vendors and in different organs.

**Figure 9:**
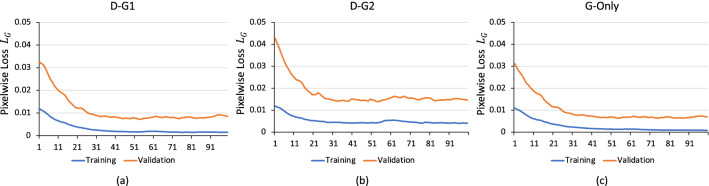
The *L*_*G*_ training and validation loss for (**a**) D-G1, (**b**) D-G2, and (**c**) G-Only training approaches. *L*_*G*_ represents the pixelwise loss between the generated image and the ground truth. The *L*_*G*_ converged to different values according to the training approaches. The G-Only has the lowest *L*_*G*_ validation loss whereas the D-G2 has the highest loss.

In ordinary GANs, the discriminator and generator networks may train at different speeds leading to an imbalanced status that may delay or even inhibit stable training convergence. Strategies have been proposed to improve this imbalance to produce faster convergence, low variance, and better image quality at early epochs^18^. Our implementation did not employ such strategies but rather continued training iterations until the generator pixelwise loss diminished and converged to a plateaued stable minimal value as will be detailed in the next sections and shown in Fig. 9. There is a need to study new architectures for the cGAN and investigate utilizing the state-of-the-art GAN models. However, eliminating classical strain calculations and reformulating the problem as a direct nonlinear mapping from the original tagged images to the corresponding strain values permits simple cGAN to provide accurate principals strain maps at native resolution. The errors produced from the proposed cGAN were substantially less than the other compared methods in error magnitude and map resolution and satisfactory from the clinical perspective since the variations in strain values within ± 0.01 are unlikely to have clinical significance.

## Conclusion

Soft-tissue principal strain maps can be measured at the pixelwise native high resolution of the raw MRI tagging images with high accuracy and precision and minimal artifacts using end-to-end Generative Adversarial Neural Network without any human interaction or preprocessing. The performance in the myocardium and the liver was consistent throughout the cardiac cycle in Monte-Carlo simulations and healthy and PAH subjects.

## Methods

### Tagging MRI

After applying two perpendicular sinusoidal line tags to an imaged section, the two tagged images at time *t* are sufficient to calculate the strain at that time frame given the tagging frequencies.

The tagging lines in direction *d* at time *t* = 0 are given by

7 $${T}_{d}\left(X\right)=\mathrm{cos}\left({2\pi f}_{d}X{{\varvec{n}}}_{{\varvec{d}}}\right),$$

where ${f}_{d}$ and ${{\varvec{n}}}_{{\varvec{d}}}$ are the tagging frequency and a unit vector in the d direction, respectively.

The tagging lines at any time *t* are given by

8 $${T}_{d}\left(x,t\right)=\mathrm{cos}\left({\omega }_{d} x\left(X,t\right)\; {{\varvec{n}}}_{{\varvec{d}}}\right),$$

and the tagged images are given by

9 $${I}_{d}\left(x,t\right)={I}_{o}\left(x,t\right) \left[ {A}_{d}\left(x,t\right)+ {A}_{T}\left(x,t\right){ T}_{d}\left(x,t\right) \right]{e}^{j{\varphi }_{e}\left(x,t\right)}.$$

Here, $$x=x(X,t)$$ as described in the background section and $${I}_{o}$$ is the effective spin density, i.e., the intensity of the anatomical image without tagging. $${\varphi }_{e}(x,t)$$ is the artifactual phase caused by susceptibility and field inhomogeneity. $${A}_{d}\left(x,t\right)$$ and $${A}_{T}\left(x,t\right)$$ represent the effect of relaxation time $${T}_{1}$$ on the effective spin density ($${I}_{o}$$) and tagging pattern ($${T}_{d})$$, respectively. After correcting for inhomogeneity, Eq. (9) can be rewritten as

10 $${I}_{d}\left(x,t\right)={I}_{do}\left(x,t\right)+{I}_{To}\left(x,t\right)\mathrm{cos}\left({\omega }_{d} \, x \, {{\varvec{n}}}_{{\varvec{d}}}\right),$$

where,

$${I}_{do}\left(x,t\right)={I}_{o}\left(x,t\right){ A}_{d}\left(x,t\right) ,$$

and

$${I}_{To}\left(x,t\right)={I}_{o}\left(x,t\right){ A}_{T}\left(x,t\right).$$

Given ${\omega }_{d}$, $x {{\varvec{n}}}_{{\varvec{d}}}$ can be estimated from the phase in ${I}_{d}\left(x,t\right)$. $x\left(X,t\right)$ can be estimated using the phases from any two tagged images ${I}_{{d}_{1}}\left(x,t\right)$ and ${I}_{{d}_{2}}\left(x,t\right)$, if ${{\varvec{n}}}_{{{\varvec{d}}}_{1}}$ and ${{\varvec{n}}}_{{{\varvec{d}}}_{2}}$ are perpendicular. Equations (1–3) show that $x\left(X,t\right)$ is sufficient to calculate the deformation gradient tensor $F$ and, accordingly, the Eulerian strains at time $t$. Due to noise, artifacts, and low MR signal in particular sites, there is no closed-form mathematical solution to extract $x\left(X,t\right)$ from the tagging images. Based on this derivation, the proposed network architecture was designed to extract the strain maps at time *t* from the corresponding two perpendicular tagging images without considering the previous time frames.

### Network architecture

Let $\tau \in {\mathcal{R}}^{N\times M\times 2}$, $\tau =({I}_{h},{I}_{v})$ represents a pair of two line-tagging images ${I}_{h}$ and ${I}_{v}$, with horizontal and vertical tagging lines, respectively, and $N$ and $M$ are the image dimensions. The images are acquired at the exact time frame $t$ and location. The corresponding Eulerian principal strains are ${E}_{p}\in {\mathcal{R}}^{N\times M\times 2}$, ${\rm E}_{p} = ({\varepsilon }_{p1}, {\varepsilon }_{p2})$ where ${\varepsilon }_{p1}$ and ${\varepsilon }_{p2}$ represent the amounts of principal stretching and contraction strains observed in the underlying imaged tissue, respectively. Let $S$ represent the function that maps $\tau$ to ${E}_{p}$, $S: \tau \to Ep$. The proposed cGAN network is tasked with finding that pixel-to-pixel mapping function $S$. The inputs to the network are $\tau$; the set of two orthogonal line tagging images and the network output is ${E}_{p}$, the associated pixel-to-pixel two Eulerian principal strains ${\varepsilon }_{p1}$ and ${\varepsilon }_{p2}$ maps.

**Figure 10:**
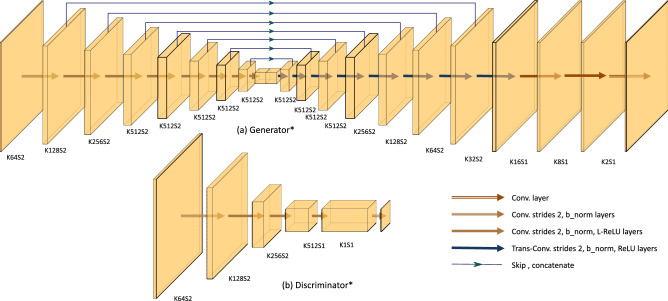
End-To-End Generative Adversarial Network architecture. K is the number of kernels, and s is the stride. The generator is shown in (**a**). The input is a 256 × 256, two-channel image composed of horizontal and vertical tagging images. The output is the corresponding two Eulerian principal strains with shapes of 256 × 256 × 2. The discriminator is shown in (**b**). The input to the discriminator constitutes the tagging images concatenated with either the generated or the ground-truth principal strain maps. *This figure is not to scale.

The generator (G) is a U-net^19^ followed by three convolutional layers, as shown in Fig. 10. All convolutional layers in the generator side used 4 × 4 sized kernels. The encoder part of the U-net includes eight convolutional layers with stride 2. The first three layers include 64, 128, and 256 feature maps, respectively. Whereas each of the last five layers includes 512 feature maps. The decoder layers include 512, 512, 512, 512, 256, 128, 64, 32 feature maps, respectively. The last three convolutional layers have 16, 8, and 2 feature maps, respectively, with stride 1. All convolutional layers in the generator except the first and last layers are followed by batch normalization and a leaky-ReLU activation function. The first layer is followed by a leaky-ReLU activation function only.

The inputs to the discriminator (D) are the tagging images concatenated with either the generated principal strains or the ground truth. The input shape is 256 × 256 × 4. The discriminator includes five convolutional layers with 4 × 4 sized kernels and 64, 128, 256, 512, and 1 feature maps. The first three layers use stride 2. Layers 2, 3, and 4 are followed by batch normalization. All layers, except the last one, utilize a leaky-ReLU activation function.

### Training approach

The original GAN loss functions are based only on the discriminator output^15^. Such loss functions are sufficient to train the network to generate images with a probability distribution close to the training image set distribution. However, some applications, such as image-to-image translation, as is our application, require generating data with desired properties. This goal could be achieved using a Conditional GAN (cGAN)^20^ framework that integrates auxiliary information during the training process. The first image-to-image translation using a cGAN was provided by Isola et al.^21^. The generator loss was a weighted sum of two losses. The first loss represented the ability of the discriminator to recognize if the generated images were fake. The second loss was the pixelwise absolute difference between the generated and the ground-truth maps. The pixelwise error contributes to generating an output map closely resembling the ground truth and does not just have a similar distribution^21–23^. Utilizing this concept, we investigated three training approaches. First, the D-Only approach uses only the discriminator's output to calculate the loss functions for both the discriminator and the generator as in the original GAN^15^. The second approach, the D-G approach, is based on the cGAN training framework^21^. The loss function of the third approach is based only on the generator's output. Hence it is called the G-Only approach. The training objective functions of the discriminator D and the generator G are set as

11 $$\underset{D}{\mathcal{L}}= \underset{D}{\mathrm{max}}{\mathbb{E}}_{{\tau ,E}_{{p}_{r}}}[\mathrm{log}\,D(\tau ,{E}_{{p}_{r}})]+ {\mathbb{E}}_{\tau ,G\left(\tau \right)}[1-\mathrm{log}\,D(\tau ,G(\tau ))] ,$$

and,

12 $$\underset{G}{\mathcal{L}}= \underset{G}{\mathrm{min}} \left\{{\alpha }_{1}{L}_{G}+ {\alpha }_{2}{L}_{D}\right\} ,$$

where,

13 $${L}_{D}= {\mathbb{E}}_{\tau ,G\left(\tau \right)}[1-\mathrm{log}D(\tau ,G(\tau ))],$$

14 $${L}_{G}= {\mathbb{E}}_{\tau ,{E}_{p}} \left[ K\parallel {E}_{p}-G\left(\tau \right){\parallel }_{1}\right],\mathrm{ and}$$

15 $$K= \kappa \parallel {E}_{p}{\parallel }_{1}+1 .$$

Here, ${L}_{G}$ represents the pixelwise loss between the generated image and the ground truth. The weight $K$ is set to moderate the effects of the low probability of observing high strain compared to low and no strain in tagging images. The value of $\kappa$ was set empirically to 10.

${\alpha }_{1}$ and ${\alpha }_{2}$ in Eq. (12) were set according to the training approach as shown in Table 1. In the first approach, D-Only, ${\alpha }_{2}$ was set to one and ${\alpha }_{1}$ was set to zero to eliminate the contribution of the generator's error ${L}_{G}$ in training $\underset{G}{\mathcal{L}}$. In the G-Only approach, ${\alpha }_{2}$ = 0 to train the generator with ${L}_{G}$ only, and thus, the discriminator was not used in training the G-Only method. ${\alpha }_{1}$ and ${\alpha }_{2}$ were both set to non-zero values in the D-G approach to utilize both ${L}_{G}$ and ${L}_{D}$. We utilized two versions of D-G; D-G1 and D-G2. ${\alpha }_{2}$ was set to 1 in both versions. In D-G1, ${\alpha }_{1}$ was set to ${10}^{4}$ to equalize the difference in magnitude between ${L}_{G}$ and ${L}_{D}$. Whereas, in D-G2, ${\alpha }_{1}$ was set to ${10}^{3}$, which increased the contribution of discriminator D in the loss function compared to D-G1 (Table 1).

Network training was performed using the Adam optimizer^24^ with an initial learning rate of 0.0002 and first and second momentums equal to 0.5 and 0.9, respectively. The network was implemented in Python and TensorFlow and was trained on an NVIDIA RTX 5000 GPU. Figure 9 shows the pixelwise loss ${L}_{G}$ for D-G1, D-G2, and G-Only training approaches during training (blue) and validation (red). Depending on the chosen approach, ${L}_{G}$ converged to different plateaued values. The D-Only method was excluded from the comparison due to its poor performance as was shown in “Results” section. The G-Only converged to the lowest ${L}_{G}$ validation loss whereas the D-G2 converged to the highest ${L}_{G}$, in agreement with both the in-vivo and simulated results above.

### Data sets

In supervised learning, network training mandates the availability of ground-truth datasets with an extensively broad range of anatomical, imaging, and deformation variations. However, it is impossible to provide the pixelwise ground-truth strain maps for the in-vivo tMRI datasets. Meanwhile, manual strain calculation by expert radiologists is a prohibitively long, tedious, and subjective process. Practically, it can only be done for a limited number of cases and at a limited number of points by which low-resolution strain maps are obtained with potentially unfavorable inter- and intra-observer agreements.

To overcome this challenge and train the proposed network appropriately, we opted to develop a versatile tMRI computational simulator. It was then used to generate highly realistic synthetic datasets with a broad range of signal-to-noise ratios, anatomical geometry, and deformation variations together with the associated ground-truth pixelwise strain maps. For performance assessment, we used another set of simulated data and in-vivo tMRI datasets from both healthy and patient subjects. We compared the GAN performances to the HARP results obtained at various bandpass filter settings, as was described in “Results” section.

### Tagging simulator

**Figure 11:**
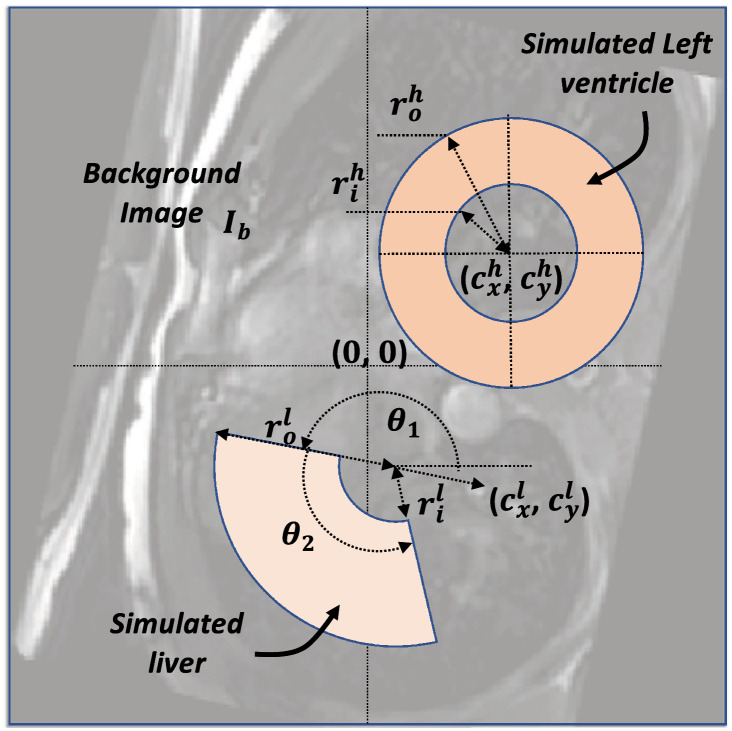
A schematic representation of the components of a simulated image. A background image ${I}_{b}$ represents the anatomical image. The simulated left ventricle and liver shapes are created based on their geomatical parameters, with values chosen randomly from their corresponding pools.

**Figure 12:**
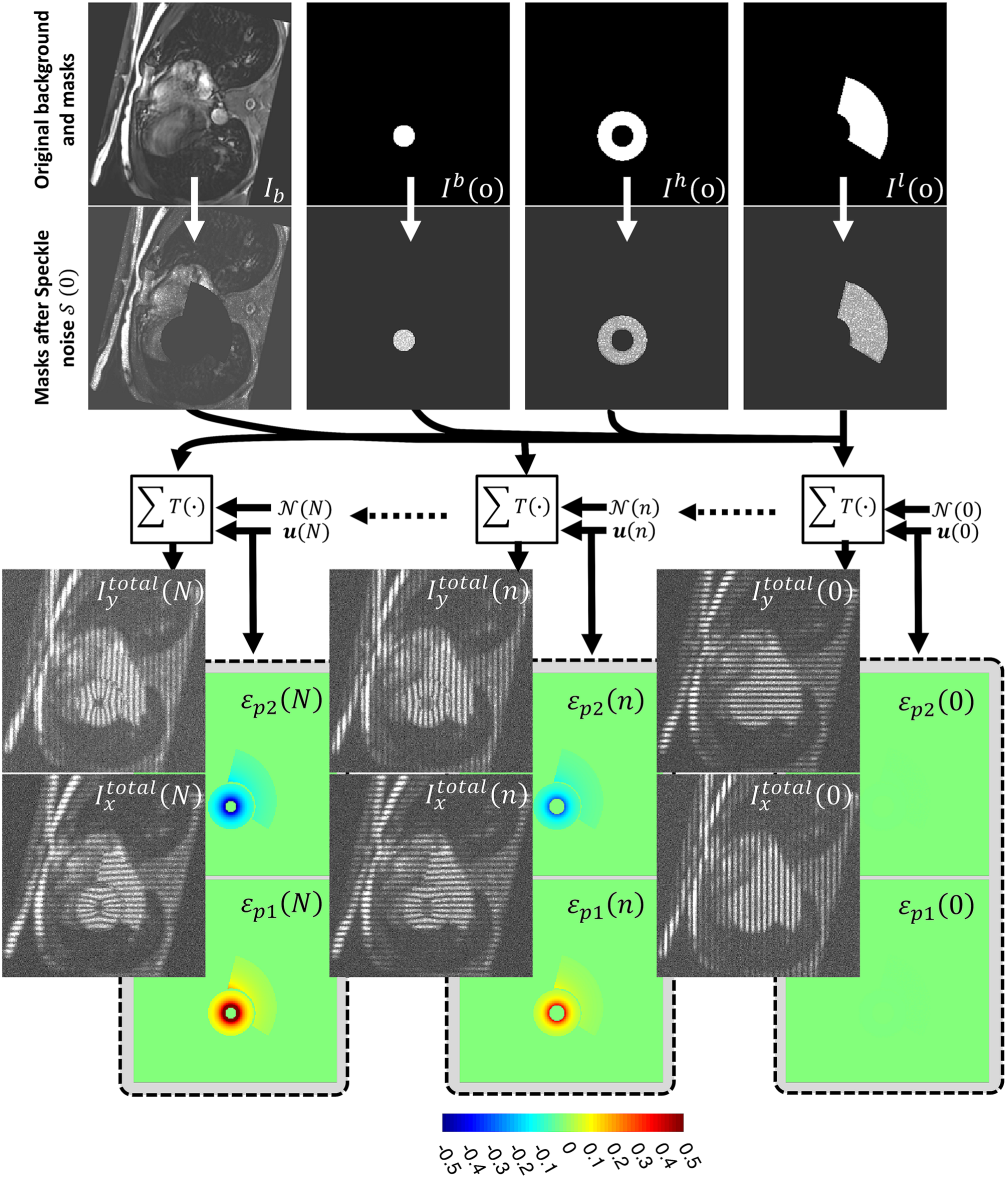
Steps of generating simulated tagging cine. A background image ${I}_{b}$ is randomly chosen from the pool of previously acquired MRI images. The anatomical and imaging parameters are then chosen from their corresponding pools. Regions of the Liver ${I}^{l}$, heart ${I}^{h}$, and blood ${I}^{b}$ are generated for all time frames following the planned anatomical and functional settings. The corresponding ground-truth strain maps ${\varepsilon }_{p1}$ and ${\varepsilon }_{p2}$ in the liver and heart are generated. Speckle pattern $\mathcal{S}$ is superimposed on the combined image. Displacement maps $u$ are then generated and used to create the corresponding tagging pattern for each time frame. Final images ${I}_{y}^{total}$ and ${I}_{x}^{total}$ are created after the multiplication of the tagging pattern and the speckled image and adding the white noise.

A computational motion simulator was developed in-house and used to simulate the tMRI cine dataset of the heart's short-axis (SA) view. Motions of the two objects LV, and the liver, were simulated while a still anatomical image ${I}_{b}$ was included as a background. ${I}_{b}$ is a randomly selected and rotated image out of a pool of 250 images previously obtained from 6 different subjects using 3D-TFE or cine bFFE sequences. The LV is simulated as a complete annulus with center (${c}_{x}^{h}$, ${c}_{y}^{h}$), and initial inner and outer radii, ${r}_{0}^{ih}$, and ${r}_{0}^{oh}$, respectively. The liver is simulated as an annulus sector with center (${c}_{x}^{l}$, ${c}_{y}^{l}$), and initial inner and outer radii, ${r}_{0}^{il}$, and ${r}_{0}^{ol}$, respectively, and angular position and width $\theta$_1_ and $\theta$_2_, respectively, as shown in Fig. 11. The values for all anatomical parameters are randomly chosen from their corresponding pools of value ranges. Figure 12 demonstrates the steps of generating a simulated tagging cine dataset.

Finally, training datasets went through a preprocessing tile-shuffling step in which the moving regions are divided into smaller regions' tiles and shuffled randomly across the image. This step is essential to train the proposed network to estimate the strains irrespective of organ shape or strain pattern. Both the original and tile-shuffled images were used for training. Six hundred unique simulator cines were generated. 4076 images from 300 cines were used for training, 679 images from 100 cines were used for validation, and 1362 images from 200 cines were used for testing.

## Supplementary Information

Supplementary Video 1.

Supplementary Video 2.

Supplementary Information 1.
