# Supplementary material for: Direct pixel to pixel principal strain mapping from tagging MRI using end to end deep convolutional neural network (DeepStrain)
Source: Sci Rep. 2021 Nov 26;11:23021. doi: 10.1038/s41598-021-02279-y (PMC8626490; doi:10.1038/s41598-021-02279-y)
Supplement: Supplementary file 3 — Supplementary Information 1. [file 41598_2021_2279_MOESM3_ESM.docx]

# Video Legends

**Healthy_Adult_Demo.mp4**

**In-vivo cine demonstration of the strain maps generated from the original tMRI cine dataset (top-left corner) by GAN-based methods (top raw group) versus HARP (bottom raw group) in a healthy subject.** Compared to HARP and throughout the cardiac cycle, G-Only GAN architecture generated high-resolution artifact-free strain maps with time-progression of strain in both right and left ventricles are perceived.

**PAH_Patient_Demo.mp4**

**In-vivo cine demonstration of the strain maps generated from the original tMRI cine dataset (top-left corner) by GAN-based methods (top raw group) versus HARP (bottom raw group) in a PAH patient.** Throughout the cardiac cycle, and compared to HARP, the G-Only GAN network could estimate the principal strain maps at the original image native resolution and without artifacts.
